# Contributions to Clinical Medicine. On Pericarditis. In Lancet from May 17, 1845, to October 31, 1846

**Published:** 1847-10

**Authors:** 


### ART. XVIII.

1. *Contributions to Clinical Medicine. On Pericarditis. In Lancet from May* 17, 1845, *to October* 31, 1846. By JOHN TAYLOR, M.D., Physician to the Huddersfield Infirmary, late Professor of Clinical Medicine in University College, London, &c.
2. *On some of the Causes of Pericarditis, especially Acute Rheumatism and Bright's Disease of the Kidneys, &c. Medico-Chirurgical Transactions,* Vol. XXVIII, 1846. By JOHN TAYLOR, M.D., &c.

IN 1826, when M. Louis produced his well-known essay on Pericarditis, it was matter of notoriety that the diagnosis of that affection was utterly unestablished. Laennec had given up the problem in despair,—he had sometimes *divined* the existence of the disease, he had never *diagnosticated* it. The inquiries of M. Louis threw no small light on the mysteries of pericarditis, as they had done on those of so many other diseases (*nihil tetigit, quod non ornavit*), and multitudes of zealous observers have, during the last twenty years, added their contributions, trifling perhaps in the state of isolation, but in that of combination forming a very imposing mass of solid information. And how, and to what degree, have the certainty and facility of the diagnosis of the disease been advanced by these labours? Where, at the moment we write, stands pericarditis in the scale of disease, which men can during life measure in their extent and in their amounts with *certainty*, facility, and dispatch? Holds it a high place, a low place, or a variable place? Were we to answer from our own experience, we should echo the term *variable*,—and affirm that pericarditis is at the present hour a diagnostic anomaly, from the facility and perfection (partaking almost of the marvellous) with which it may in all its forms, offsets, and minutest modifications be diagnosticated in some instances, while in others (even where the disease is fully developed, and where it is looked for) it escapes detection altogether. In the present place it is enough to state this proposition; the opportunity for illustrating it will occur more than once in connexion with various facts which we shall presently be called upon to pass in review.

But if the historian of pericarditis may find himself (presuming him to be a specimen of that too rare character—an *honest* medical historian) loading his page perforce with numerous names — names of the first noticers of this murmur and of that bulge, of this ache and that palpitation—he will unquestionably, on the other hand, discover that to the name of a single individual alone may legitimately be attached the honour of having thoroughly worked out the disease in all its phases and bearings—and that individual is Dr. Taylor. Dr. Taylor, having been for a considerable number of years closely engaged in clinical observation, found himself at length possessed of a mass of recorded cases of various maladies, and deemed the time had come when a profitable analysis of the whole might be made. He selected pericarditis as the disease to commence with, and after due consideration of the guise in which he might most profitably appear before the public, fixed upon the course of proceeding adopted in the contributions we are about to notice. He determined upon publishing separately all the cases of the disease which he had recorded, accompanied with comments upon their leading features,—the collection to be followed by a numerical analysis of the whole, adapted to exhibit the inferences fairly deducible therefrom in respect to the symptoms, diagnosis, causes, and other leading phenomena of the disease. Now there are various reasons which led Dr. Taylor (and others who, like ourselves, agree with him) to believe that this method of *publishing* the details in full of cases, which are to serve as the basis of general inferences, the only just and proper one—where it *can* be pursued. In the first place, conclusions drawn from the review of a number of cases, instead of being taken upon trust, are capable of being verified; and additional questions, such as never occurred to the author, may be answered by other persons who may subsequently engage in similar investigations. Secondly, the character of the cases will furnish a test of the confidence to be placed in the man who records them, and would teach us by their analysis: there will of necessity appear in their narratives evidence of his honesty or dishonesty, his fitness or unfitness to observe, &c. In the commentary on each individual case, there is, in the third place, an opportunity afforded of introducing observations on numerous subsidiary points, which could not so well be made the subjects of consideration in a general analysis. Many other sound reasons (and others yet further we could ourselves urge) may be found in Dr. Taylor's pages exhibitory of the importance of full detail in the published narratives of cases. But it is useless to dwell on these; the broad fact is this, that true practical medicine being confessedly capable of sure advancement by but one solitary means (slowly, ever, it is true), namely, by minute observation and numerical analysis, the public display of the elements of that analysis cannot be too constantly nor too completely effected.

The cases of pericarditis, which Dr. Taylor has published, are forty in number; all (with two exceptions) are accompanied with commentaries such as just alluded to, but no *analysis* of the whole has yet been printed by their author. Our task, therefore, in working out some of the results to which these cases lead, will be by no means a light one; but in the interest of our readers we cheerfully enter on the labour.

*Friction-sound.* It is a fact familiarly known that pericardial friction-sound may very closely simulate valvular murmur, and several examples of the difficulty of distinguishing the two things occur in Dr. Taylor's cases. Indeed Dr. Taylor admits that there are conditions under which he has been unable to determine the true seat of the morbid sound; thus we find him in one instance saying: "On Nov. 12th and 25th, the characters of the sound at the base of the heart were such, that it might have arisen either from some slight remains of the pericarditis, or from a trifling amount of inflammation supervening in the aortic valves. If the cause last named were the true one, then it is worthy of remark that the sound was not (probably on account of its feeble intensity) propagated into the arteries of the neck." In another case, where the *character* of a "double, continuous, rough murmur" was considered insufficient to localize it in the pericardium, rather than in the endocardium, its pericardial origin was inferred from the facts that "it was heard extensively, and loudest at first where valvular sounds are seldom loudest (fifth left costal cartilage), and afterwards changed its point of greatest intensity to opposite the third rib. This fact agreed best with the notion that the pericardium was the seat of the sound, and seemed to indicate either adhesion or, more likely, greater serous effusion." Besides "the morbid sound was very loud over the third rib, and scarcely audible at the top of the sternum—a circumstance which could hardly occur in aortic-valve disease." The event proved the correctness of the diagnosis. But there was in this case another remarkable circumstance, "a single rough bellows-sound, synchronous with the pulse, audible along each side of the vertebral column in the greater part of its length, but loudest on the right side opposite the fifth and sixth dorsal spines." Dr. Taylor thinks that this sound originated in the pericardium and not in the aorta, for the following reasons: "1. It was loudest about the fifth and sixth vertebræ, and therefore opposite to the heart. 2. The great *transverse* extent of the sound, without any evidence of disease of the lung to conduct it. 3. Its diminution (on the 24th of the month) when the friction-sound in front diminished, and when there was evidence of increased serous effusion into the pericardium. 4. Its cessation with the friction-sound on the 30th." A difficulty on the face of this explanation is that this sound behind was *single*, while that in front was *double*. "Perhaps," suggests Dr. Taylor, "the single sound depended on a fibrinous exudation on the posterior surface of the pericardium, so disposed as to occasion a friction-sound chiefly with the ventricular systole; considered in itself, the sound resembled that occurring in disease of the aorta, much more than the rubbing sound of pericarditis; but independently of the considerations adduced above, it may be presumed that it would have been less likely to cease in the former than in the latter case."

The *creaking* quality sometimes assumed by pericardial friction-sound, has been commonly set down as significant of stretching of adhesions. Cases collected by Dr. Taylor exhibit the error of this notion. In one of these "the serous surface of the pericardium was everywhere covered with soft lymph, and there was no point of adhesion. The roughness of this lymph was so trifling, as to be barely visible, but it was most distinctly appreciable by the finger passed over it." In another instance, at the very time that friction-sound became *creaking* in character, there were noticed signs of a moderate amount of serous effusion into the pericardium. From this it would follow that *much pressure* of the opposed surfaces is not required for the generation of this particular variety of rubbing sound.

Dr. Taylor refers to but one example of the *crumpling* variety of this sound; it was here superficial, diffused nearly over the entire cardiac region, and lasted about twenty-one days.

The full development of friction-sound does not seem readily possible, unless both divisions of the pericardium are the seat of plastic exudation. A case appears among those set down by Dr. Taylor, in which no pericarditis was suspected during life, yet the "whole surface of the heart was covered with recent lymph," while the loose division of the pericardium was totally free from it. Now the heart had been examined during life, and a "trifling murmur with the first sound at the base" discovered. This was considered to be an obstructive aortic murmur; but the post-mortem examination proved that the aortic valves were healthy, and the author does not appear disposed to regard the murmur as anemic. Hence we would draw the inferences from this case that: 1st. Pericardial friction-sound (single and systolic) may be produced, but not to any great amount, by the rubbing of lymph limited to the cardiac division of the membrane, and this in the very situation (namely, at the origin of the great vessels) where motion exists in the least marked degree. 2d. Such friction-sound may be readily confounded with a systolic aortic murmur; means of distinction may readily be imagined, but as none were tested in the case, we will leave them all unstated. Now, in the inference which we have just drawn (No. 1), we are at variance somewhat with the spirit of Dr. Taylor's remarks on this point: his inclination is rather to insist on the source of fallacy arising from the almost total deficiency of friction-sound, when only one division of the pericardium is the seat of lymph-exudation. Perhaps this is the manner of viewing the point which is best calculated, generally speaking, to be practically useful; because it will teach the observer to abstain from a positive negation of the existence of pericarditis on the evidence of imperfect or (presumedly) deficient friction-sound. But, on the other hand, it is clear that the *ultimate truth* is such as we have stated it, and to *this* the clinical investigator should certainly point his aim.

Several of Dr. Taylor's cases prove the fact that a considerable amount of serous infusion does not necessarily prevent the occurrence of friction-sound at the same time. In one of these cases the rubbing sound was heard on the day of the patient's death, and eight ounces of serum were found in the pericardium.

In one instance friction-sound existed, disappeared, and returned again four days later. This disappearance of the friction-sound could not be ascribed to serous effusion, as no increase in extent of the heart's dullness could be detected. The return of it coexisted with an exacerbation of the rheumatism, under which the patient laboured at the same time.

*Heart-sounds.* The feebleness and deficiency of tone in the heart's sounds, well known to accompany serous effusion into the pericardium, are presumed to depend solely upon the deadening influence of the fluid enclosing the heart. But a case noticed by Dr. Taylor at least opens the question whether these alterations of the natural sounds may not depend, at all events in part, upon some coexistent condition. In this instance the alterations in question were noticed, though little or no serous effusion was present. It is suggested that "the first sound might be diminished by some modification in the contraction of the muscular fibres of the left ventricle, consequent on the adjacent inflammation, or, at least, as much of the sound as depends upon muscular contraction; or both the sounds might exist, but be marked by the loud friction-sound."

*Cardiac dullness.* In various parts of his commentaries, Dr. Taylor insists very emphatically on the importance of percussion-dullness in the cardiac region as a sign of effusion. He observes very justly that its diagnostic importance is extensive, first, on account of the large number of cases in which it occurs; and secondly, because this sign is readily appreciable, and not unfrequently constitutes the only physical evidence of the disease to be found at the time of observation (that is, without waiting to watch the *progress* of the disease). But hypertrophy causes dullness also; how are the two kinds to be distinguished? Dr. Taylor replies that in the instance of pericardial effusion: "1, The dullness extends *upwards* to the level of the second rib or clavicle, and but little below the healthy limits *downwards;* 2, It extends further to the *right* side than that of hypertrophy of the *left* ventricle, and further to the *left* side than that of hypertrophy of the *right* ventricle; 3, It changes in its extent from day to day, and sometimes rapidly." These characteristics are more clearly and unmistakeably put by Dr. Taylor than by any other writer.

The dullness of condensed apex of the left lung, might be confounded with that of pericardial effusion. In a case illustrating this matter, the dullness was pronounced to be pulmonary, because, "although it extended up to the clavicle, yet it was *complete* only up to the third rib (as high as the heart reached); and *incomplete* above that point, as if the sign in these two situations depended upon two different causes; moreover, the transverse extent of the dullness was not so great as it ought to be in pericardial effusion of such amount as to reach the clavicle—its extension was less towards the right of the cardiac region (being more prolonged towards the left side, as in hypertrophy of the left ventricle), and it did not vary in extent from time to time." The diagnosis was confirmed by the fact that the dullness persisted after the cure of the pericarditis.

Two cases are referred to as proving the production of percussion-dullness over the upper and *back* part of the left side of the chest by pericardial effusion. In both of these, however, the left lung was in a condition to make it doubtful whether this might not have been the true cause of the dullness; at all events the narrations are not convincing.

*Heart's impulse.* The rule is, that where serous effusion exists, especially to any amount, in the pericardium, the heart's impulse becomes deficient in force. This rule would appear to suffer occasional exceptions; in one of the cases before us, in particular, it does not appear that any peculiarity was remarked in the impulse of the heart, notwithstanding the existence of copious serous effusion. Certainly no mention of diminution of impulse is made in the report of the case; but it would have been more satisfactory, had the natural state of the heart's impulse been made matter of affirmation. We do not ourselves remember to have seen effusion without modified impulse; and it is to be borne in mind that a positive opinion cannot be fairly acquired, unless when the patient has been examined both *before* and *after* the serous fluid has been accumulated. Dr. Taylor did not see the subject of the case in question (No. xix) until after the pericardial sac had undergone great distension with serum.

*Undulatory movement.* Much has been written and speculated concerning "undulatory, or vermicular motion" accompanying the impulse in the cardiac region. Dr. Taylor's cases illustrate its occurrence in connexion with serous effusion, with enlargement of the heart, where no serous effusion exists, and with adhesion of the pericardium. Hence, they who maintain that such motion is specially significant of adhesion alone, err grossly: we have, within the last few days, observed one of the best specimens we have ever seen of this said vermiculation, in a case of pericarditis with serous effusion insufficient in quantity to materially alter the quality of the friction-sound. Much nonsense has indeed been put forth from high places concerning this matter; and we have more than once had evidence at the bed-side of one of its probable causes. People's minds are not made up as to what constitutes vermicular movement: some persons scarcely ever look at a cardiac region without fancying they see an example of it; others cannot perceive it where it absolutely and fully exists.

*Tactile vibration.* It is shown that vibratory tremor, when occurring at all, is of shorter duration than the accompanying friction-sound: a more powerful rubbing is required to render friction of membranes a tactile than an audible phenomenon. Tactile vibration is modified in amount, but not necessarily destroyed, by the occurrence of serous effusion. Where valvular disease coexists with pericarditis, and vibratile tremor is discovered, it may be difficult, on first view, to determine to which affection the tremor is due. But, if pericardial, it will accompany both movements of the heart, and will be coextensive with the friction-sound: temporary duration of the tremor will eventually prove the diagnosis. Vibratile tremor may exist in one and the same cardiac region, both of valvular and of pericardial origin; and it may be possible to distinguish them under favorable circumstances. Thus, in a case of Dr. Taylor's, a tremor existed at the apex in connexion with mitral regurgitation, and for some time was the only vibration to be felt. A month later the base became the seat of tremor in connexion with an attack of pericarditis; the friction-sound of this affection remaining after the new tremor had disappeared. But here the diagnosis was made through an *accident* of localization; suppose the pericardial tremor had arisen at the apex, how would it have been distinguished from the mitral, to which it was superadded? The distinction would have been impossible in the existing state of knowledge.

*Cardiac bulging.* Among the physical signs of pericarditis, revealed to observers in the essay of M. Louis, appeared bulging or vaulted form of the cardiac region. M. Louis ascribed it to the influence of serous effusion, believing that, previous to the occurrence of such effusion, it cannot exist. M. Gendrin has more recently attempted to show that the alteration of form in reality depends on paralysis (through adjacent inflammation) of the intercostal muscles, and may arise previous to effusion, and constitute one of the very earliest signs of pericarditis. We have looked carefully, in a certain number of instances, for evidence in support of M. Gendrin's view, and have as yet failed to discover it. On the other hand, too, it is certain that a good amount of effusion may exist without inducing any precordial bulging,—we know this from our own experience, and Dr. Taylor's Case xix gives additional demonstration of the fact. Further, hypertrophy of the heart is, according to our observation, a much more frequent cause of cardiac bulging than serous effusion. From all this, the inference follows that the absence of bulging is not to be looked on as affecting the diagnosis of effusion, nor is its presence to be considered confirmatory of the existence of such effusion in a doubtful case, unless the bulk of the heart itself has been, within a short period, ascertainably natural.

*Failure of diagnosis.* The importance of the physical signs, which we have now, under Dr. Taylor's guidance, considered in certain of their characters, cannot be exaggerated. If this be esteemed too strong a statement, let us simply refer him, who thus judges it, to the fact that among Dr. Taylor's *forty* cases were *fifteen* at least in which the disease was revealed by these signs alone. On the other hand, precise and distinctive as these signs are, and easily as they are to be discovered, except under a concourse of peculiarly adverse circumstances, it is an unfortunate truth that pericarditis escapes detection occasionally during life; in *ten* of Dr. Taylor's forty *cases,* the existence of pericarditis was either not suspected, or, at least, not substantiated. Let us inquire into the reasons of this. The first general character traceable in these cases of failure of diagnosis is that they were all cases of *non-rheumatic* pericarditis,—hence of pericarditis arising under circumstances presumed to be less usual than certain others,—hence of pericarditis likely enough not to be inquired after or looked for by the physician. Was this non-inquiry after the disease, then, the invariable cause of its escaping detection? *A priori,* we should fear not (and the reason why we use the word *fear* is obvious), for Dr. Taylor frequently makes incidental reference to the fact that it is his habit to examine all the organs of individuals placed under his care. We should further fear not, because we must confess we have ourselves twice failed to detect the disease (under peculiar physical conditions of the individual, and under peculiar combinations of functional derangements of organs unconnected with the heart), though we had examined the cardiac region not uncarefully, and if not precisely under the impression of probably finding pericarditis, at least not under the stultifying influence of any preconceived idea of its absence. What, then, actually were the peculiarities of these ten cases of Dr. Taylor?

It appears, in the first place, that three of the ten were not seen by Dr. Taylor; the cases we have to account for, therefore stand as seven out of thirty-seven, and in all but one of these seven the cardiac region was examined more or less carefully during life. In the first of these (Case xxx) the pericardium was universally adherent by means of *recent* lymph, and this state of adhesion existed at the period of the patient's admission. In the total absence of *symptoms* to direct attention to the heart, the detection of this condition of the pericardium was utterly impossible; or rather it would have been impossible to affirm, admitting that the adhesion had been detected, to have pronounced this to have been of *recent* rather than of more or less *distant* origin. In Case xxxi there were ten ounces of bloody serum in the pericardial sac, and nearly the entire of both surfaces, cardiac and parietal, were covered with scabrous lymph. The patient in this case was admitted with the effusion, therefore no friction-sound was detected, though sought for. Dullness more extensive than natural existed in the cardiac region, but its characters were not carefully inquired into. The man had hypertrophy of the heart also; but no symptoms directing attention to that organ. Here, then, the observer's faculties were especially wakened to the possibility of the existence of pericarditis, yet in its most intense form it eluded discovery. In Case xxxii there were twelve ounces of serum in the sac, the walls of which were besides coated with soft lymph. Here no examination of the cardiac region had been made during life. In Case xxxv, the cause of failure was the same as in Case xxx; the laminæ of the pericardium were universally adherent at the time of the man's admission. In Case xxxvi the pericardium was greatly thickened; there were old adhesions in front, and at the back of the left ventricle, and at the apex; also recent adhesions on the right ventricle, while the remaining cavity held serum and a solid loose fibrinous coagulum. The subject of this case was an old man dying of vesical disease, and it seems possible that the pericarditis may have occurred during the several weeks preceding death, and during which (attention being engrossed by the urinary disease) the cardiac region was not examined. Or, on the other hand, it may be conjectured that the heart having really been examined, no friction-sound was detected,—or (it may be) produced, on account of the peculiar anatomical condition of the diseased sac. In Case xxxix friction-sound was mistaken for valvular murmur, the examination being carelessly conducted. In Case xl the pericardium contained about a pint and a half of flocculent serum. In this instance the patient had been transferred from the surgical wards for an attack of pneumonia occurring while he was under treatment for an injury to the knee. The diagnosis had been as it were settled, when the boy (for such he was) reached the medical wards, and was quietly accepted; the more readily as he was so excessively ill-tempered that force was required to make him submit to auscultation.

Looking then to these cases, we may draw the general inference that failures in the diagnosis of pericarditis are to be referred either to careless examination, or to the fact that either adhesion or abundant effusion has already taken place when the case falls first under observation,—each of these causes of failure being as it were collaterally supported by the total absence of cardiac *symptoms*. When adhesion has occurred at the period of the patient's admission, we believe it impossible to determine the fact of its *recent* origin (admitting that its actual existence has been satisfactorily made out at all); especially as there will in all probability be quite enough in the lungs, pleura, or elsewhere, to account for any acuteness in the character of the disease. As respects the detection of pericardial effusion developed fully at the period a patient, whose commemorative history is imperfect, and who labours under other disease also, first comes before us, we confess that (while we agree with Dr. Taylor as to the great significance of the *form* of the percussion-dullness, when the peculiarity of that form is well developed) positive affirmation does not appear to us, no matter how careful the examination, to be *always* possible or justifiable.

There are a certain number of cases in which all physical signs of pericarditis are deficient, though the anatomical state, apparently of necessity productive of some of them, is found after death. Three such cases appear in Dr. Taylor's collection, and each is referable to a different category. In one the deficiency of signs arose from the interfering influence of old adhesions of the pericardium. Upon this source of difficulty we have had occasion to comment in a former number (Vol. XXIII, p. 172, January 1847) when engaged in the examination of Dr. Latham's antithetic phrases on the subject. We then took occasion to observe that though persuaded (as matter of common sense as well as of experience) that when old adhesions are close and universal, a new inflammation will give no physical evidence of its existence, we were equally persuaded from *actual observation* that a new pericarditis may, in spite of the past existence of an old one, give rise to sound referable to friction of the serous laminæ. And this for the simple reason that *complete* adhesion is not the invariable condition of a pericardium which has gone favorably through an attack of inflammation; *incomplete* adhesion (such adhesion as shall not utterly prevent gliding motion) is the rule. The murmur generated in such a *spoiled* pericardium may be deficient in true *friction* character it is true; but, as we have before said, "the position in which the murmur is heard, its instability in character as in precise locality, its non-transmission beyond the precordial region, its evidently superficial seat, —all these conditions will point to the pericardium as the seat of the new-made sound." And further (supported by experience acquired since the above sentence was first written) we would add (what does not appear to have been noticed by any of the describers of pericarditis), that although the quality of *friction* or of *creaking* may be utterly wanting in a given murmur audible in the cardiac region, still certain peculiarities in its *special character* may (quite independently of considerations of localization, propagation, &c.) suffice to fix the pericardium as its seat. In Dr. Taylor's second case of deficiency of friction-signs, their absence appears to have in all probability depended on the limitation of *recent* exudation to the posterior surface of the heart; the existence of old adhesions in front of the organ, may, however, have also played its part in the same direction. In the third case, lymph was effused on one only of the two surfaces of the pericardium; possibly the unaltered smoothness of the other surface may have prevented friction-sound; but the heart's action was here very feeble likewise.

*Symptoms.* We may now turn to the review of certain functional results of pericarditis. Orthopnœa is one of those concerning which writers have dwelt most complacently; and its existence, in connexion especially with tendency to syncope, immobility of the trunk, &c., has been set down as distinguishing cases attended with liquid effusion from others in which plastic lymph had alone been thrown out. Now we know from our own observation that this doctrine will not hold good at the bed-side; and Dr. Taylor supplies (in one direction at least) very precise and gratifying destruction of its provisions. In the first place, he gives seven cases in which there was liquid effusion without orthopnœa; in one of the number there were coexistent enlargement of the heart and pleural effusion. In the second place three cases are recorded in which there was orthopnœa, though no serous effusion had occurred in the pericardium. In the third place appear two singular cases in which orthopnœa set in after, and not until after, the absorption of serum in the sac. In these latter cases Dr. Taylor suggests that the orthopnœa (which was paroxysmal and accompanied in one of them by spasmodic cough) was referable to the "irritative influence of the inflammation in the heart or lungs propagated to the spinal cord, and reflected to the lungs again." The orthopnœa and cough, under such circumstances, appear, he continues, "to belong to the same order of symptoms with the chorea-like motions of the limbs in other cases."

In connexion with this view of the occasional nature of the orthopnœa of pericarditis, it may be well to refer to Dr. Taylor's notions on the subject of spasmodic movements in pericarditis generally. As is well-known, numerous cases are on record in which individuals, having presented distinct apoplectic or epileptic symptoms, maniacal symptoms, tetanus or trismus, chorea or aggravated hysterical attacks, have been examined after death, and found in possession of healthy brains and medullæ. In all of these cases the pericardium was actually inflamed; in half at least of them with rheumatism, and in the rest without rheumatism. Now it is matter of accredited doctrine that in such cases a certain influence is transmitted from the inflamed pericardium "to the nervous centres, and reflected upon the motor roots of various parts." In commenting upon a case of pericarditis attended with such phenomena, Dr. Taylor speaks as follows:

"The case before us shows that a different explanation of some of these cases may be suggested and supported, I think, by at least an equal amount of evidence. 1. There had been cramps in the legs, and occasional loss of memory and double vision, before the pericarditis appeared; this disease could not, therefore, be the cause of the symptoms in question. 2. The coma and other recent nervous symptoms would all have been referred, without hesitation, to the renal disease" [Bright's disease existed in the case], "if no pericarditis had been found. 3. I have elsewhere (Med.-Chir. Trans. vol. xxviii) attempted to show that the mass of cases of non-rheumatic pericarditis is due to renal disease. 4. I believe it will be found that a large proportion of cases of non-rheumatic pericarditis is quite latent, in the ordinary acceptation of the term, and also that the mortality is much greater than among the cases of rheumatic pericarditis. I therefore regard the coma and the pericarditis, in this case, as the common effects of the renal disease, and I believe them to be altogether independent of each other. I further believe it to be probable that the pericarditis and the nervous symptoms stand in the same relation to each other in a certain proportion of the published cases referred to. One or two particulars may be pointed out, either confirmatory of this view, or consistent with it. (1). In no one of the cases collected by Dr. G. Burrows (Med. Gaz. May, 1843) has the state of the kidneys been expressly mentioned after death, or the presence or absence of albumen been noted during life. In several of these, indeed, it is stated that there was no morbid appearance in any other organ than the pericardium, but all who are practically acquainted with the way in which bodies are inspected, and the appearances recorded, will know that such a statement affords hardly the least proof that the kidneys were even seen, much less that they were so examined that they might be pronounced healthy. (2). The mortality was twice as great in the non-rheumatic as in the rheumatic cases" [collected by Dr. Burrows]; "in which respect the two classes resemble, respectively, cases of rheumatic and renal pericarditis. (3). All the cases with apoplectic symptoms occurred among those without rheumatism; and one of the patients is stated to have had general dropsy. One of the rheumatic cases having ended in coma. Two of the three tetanic cases belonged to the non-rheumatic class. From this comparison, therefore, of my own cases of renal pericarditis, and those of non-rheumatic pericarditis, I think there is much probability in the supposition that the kidneys were diseased in some, at least, of the cases of the latter class."

We have transferred this passage in full to our pages, because it not only refers to a most important point, and one concerning which we perfectly and fully coincide with the learned writer, but because it exhibits a fair specimen of Dr. Taylor's lucid and simple, unpretending style, and of his close, logical, and conscientious mode of solving clinical problems.

To the class of spasmodic symptoms Dr. Taylor refers great irregularity of the pulse (at one time 70, at another 120-130, and subsequently not to be counted, from its indistinctness and irregularity); presuming it to arise from "spasm of the heart, analogous to spasm of other muscles." To this notion it may be objected that pain is a concomitant of spasm in muscle generally, that there is no reason why it should not be present in spasm of the heart (and, indeed, some excellent observers hold that the essential and sufficient cause of the paroxysm of angina pectoris is none other than this same cardiac spasm), while in the instance referred to there was none present. The heart in this case was excessively soft and dilated,—a state of things which has been found consistent with frequent and irregular pulse under various circumstances; but it of course remains to be determined in what manner such softness generates the peculiar condition of the heart's action.

Taking Dr. Taylor's cases together, the pulse is found to have been very often regular, sometimes rather full and jerking, sometimes irregular or intermittent for a time, and sometimes thready. Thread-like character of the pulse, observes Dr. Taylor, "I suppose to be due to the mechanical compression of the left ventricle (by effused serum), and the consequent obstacle offered to the free entrance of blood into its cavity, as well as perhaps to the free contraction of the muscular fibres. . . . . In respect to its diagnostic value, however, I may remark, that I have observed cases of pericarditis with serous effusion, in which this smallness of pulse was not present; and also, that the same kind of pulse is of frequent occurrence in various organic diseases of the heart without any pericarditis, as well as in other diseases not implicating the heart at all, except functionally." Then why, it may be asked, not consider it a functional, rather than a mechanical effect of pericarditis, where it accompanies this disease?

Remarking upon the occurrence of sudamina in the last stage of a case of fatal pericarditis, and the alleged connexion of sudamina generally with profuse perspiration, Dr. Taylor observes that, "in the present case no eruption was noticed when the sweat was profuse; and when the former did appear, the perspirations had ceased to be remarked. It was most abundant near to the blistered surface, as if the local irritation had something to do with the production of it." He is disposed, with the majority of persons, to refer the occurrence of sudamina to some peculiar condition of the system.

The formation of adhesions of the pericardium (or of agglutination of the two laminæ) is illustrated by several of Dr. Taylor's cases—five at least. From the remarks on these cases we learn that the speaker calculates on fixed position of the heart's apex and undulatory movement (especially if this be very well marked and constant) as diagnostic of the anatomical condition in question. But such diagnoses are generally to be made on the dead subject only. It has been our fortune to meet with two individuals possessed in their own opinions of a peculiar faculty for the diagnosis of "adherent pericardium;" and they were certainly both remarkably successful. Their success, however (it may be as well to add), uniformly consisted in detecting adhesions when the post-mortem examination proved them to be absent, and in failing to discover them where they really existed. There is one point ingeniously and neatly made out by Dr. Taylor in connexion with this matter, which we may not pass over without notice. It would seem that where hypertrophy (or rather enlargement generally) of the heart co-exists with pericardial agglutination, the existence of the latter modifies (or may modify) the position of the heart in such manner as to enlighten its own diagnosis. In commenting on a case of the kind, the Professor observes: "Notwithstanding the great volume of the heart, the apex was not lower than usual, nor further towards the left side, and the base was something higher. This is certainly not what generally happens in enlargement of the heart without adhesion of the pericardium, in most of which cases the apex is either depressed or displaced laterally, or both." Now this seems a fitting place to put a problem in diagnosis to Dr. Taylor, to which we should be glad to have his answer. Given, a middle-aged subject, stupid and unable to supply a trustworthy account of himself, even as to the precise time he has been suffering, evidently labouring under some affection of the heart or its appendages, which affection is unattended with local pain or palpitation (or, in a word, with any symptom connected by the patient himself with the cardiac region); the subject is flabbily and abundantly fat in person; the impulse of the heart is very feeble; as well as can be distinguished (for this is very obscure), the apex-beat corresponds to the upper edge or posterior surface of the sixth rib on the vertical level of the nipple; the precordial dullness is considerably more extensive than natural, and at the base rises above the left third cartilage, reaching the second intercostal space certainly, and possibly [with two ? ?] in a minor degree the second left cartilage; the heart's sounds are feeble and toneless; there is neither endocardial nor pericardial murmur, and undulatory movement cannot be traced. Now, here is a picture (which we know Dr. Taylor's practised mind will at once recognise as clinical, real, and unexaggerated in its difficulties), and we ask him how he would, under such circumstances, determine whether the patient was the subject, on the one hand, of soft, flabby (it may be fatty) enlargement of the heart, combined with agglutinated pericardium, or, on the other, of more or less recent hydro-pericarditis? Dr. Taylor may say, the form of the dullness will settle the point; if so, we demur: for we deliberately affirm that, when an enlarged heart is adherent, not unfrequently will its dullness be found to extend upwards quasi-pyramidically in most puzzling propinquity to the second left cartilage. In rare cases the same difficulty will arise (probably through a loaded state of the right auricle especially) independently of the existence of adhesion. Again, it may be said, that were the case one of hydro-pericarditis, the obscure apex-beat would be discovered on a higher level than that supposed. Now we are quite aware (for we have more than once seen the thing) that it is amongst the tendencies of accumulation of liquid in the pericardium to twist the apex somewhat upwards, and so *raise*, instead of depress, the point of the apex-beat: but this is a physical sign, to the absence or contradiction of which we are positive Dr. Taylor would agree with us in not attaching any serious importance, so uncertain is its signification in (at all events) the present state of knowledge.

*Course of the disease.* The fact that pericarditis occasionally undergoes temporary suspension in its course receives full illustration from the records of Dr. Taylor. One case is referred to where circumstances arose lending some plausibility to the notion that a suspension of twenty-three days' duration occurred; but these circumstances, we would observe, do not altogether exclude another and more simple explanation.

The morbid changes which pericarditis, cured symptomatically, inflicts upon the heart itself, are by no means to be despised. The chief are (as enumerated by Dr. Taylor) hypertrophy of the left ventricle, dilatation with hypertrophy of the same ventricle, and atrophy of the heart generally. In commenting upon a case where great dilatation of the left ventricle co-existed with comparatively very slight hypertrophy, he observes: "In the present case I see no cause for the great dilatation, compared with the thickness of the ventricle; but the close adhesion of the pericardium to the heart and to the ribs would tend to prevent the walls of the ventricle from approximating towards the centre of the cavity. This supposition, however, would lead us to expect some, if not an equal, degree of dilatation, of the right ventricle, which yet was not observed to exist." Not having ourselves distinctly seen atrophy of the heart as a consequence of pericarditis (under circumstances when the pericarditis alone could have been its cause) we turned with considerable interest to Dr. Taylor's evidence and commentary thereon. But his evidence is deficient beyond all question. Other persons have published statements of the same character as Dr. Taylor's; but it has, on the other hand, been affirmed by certain pathologists (crotchety ones, it may be, but withal experienced) that cardiac atrophy is never produced by the embrace of pericardial false membranes.

Among the morbid appearances produced or left in the pericardium itself there are some which Dr. Taylor illustrates in a manner requiring notice.

Thus, in a case of pericarditis, apparently very rapidly fatal, "the pericardium externally was quite dry and was transparent, like oiled-silk;" and the writer observes, that he has repeatedly met with this condition without being able satisfactorily to explain its mode of production. Dryness, however, as an early effect of inflammation of membranes is a well-known condition in those of the serous class; it is the cause of the earliest physical sign (grazing friction-sound) of pleurisy, and is frequently observed in the arachnoid. In membranes of the mucous class its occurrence is exemplified by coryza. It is certainly curious, however, that in the case of dry pericardium particularly referred to now (No. vii), Dr. Taylor is disposed to believe that there had been tolerably abundant effusion present during life. No such effusion was nevertheless found (two drachms constituted the whole amount); Dr. Taylor supposing that it had been absorbed during the twenty-four hours preceding death.

The author's Case xxvii gives him an opportunity of discussing the question whether pericarditis is curable without adhesions. In this instance the signs of that inflammation existed during life; the post-mortem inspection revealed "not more than three or four drachms of serum in the pericardium, and that not turbid; great redness of the serous or subserous tissue of the visceral pericardium, a little over the anterior, but chiefly over the posterior, surface of the heart; the redness was composed of fine blood-vessels, and was not uniform as in mere staining by imbibition; no loss of smoothness or transparency; a few spots of rather firm false membrane appeared in the course of the anterior coronary artery, easily scraped off with a scalpel." Now upon these conditions the lecturer thus comments:

"Looking at the morbid appearances apart from the symptoms, it might be disputed whether inflammation of the pericardium had existed. The redness, however, had the characters of that produced by inflammation, as far as such characters can be considered to be distinctive. The unequivocal double friction-sound, with nothing else to explain it, appears a decisive argument in favour of the same view. The friction-sound, then, may be produced by inflammation accompanied by mere redness and without effusion of lymph, or otherwise the inflammation must have been in process of cure, and the effused lymph had been absorbed. Seeing that the sound had disappeared for several days before death, it is probable that the latter is the true view. Upon either supposition the case proves that pericarditis, attended with a friction-sound, may be cured without any adhesions, and probably without any other traces of the disease existing."

The modes of death dwelt upon by Dr. Taylor as distinctly connected with the nature of the disease are by sudden suspension of the heart's action and by gradual failure in the power of the left ventricle. Of the former mode of death, not uncommon in heart diseases generally, Case xix furnishes a very distinct and instructive example. In this instance, as in others which have fallen under our own notice, the sudden cessation of the heart's contractions was caused by abrupt change of position on the part of the patient.

The post-mortem examinations of Dr. Taylor are recorded with such care and minute accuracy, that their analysis could not fail to throw great light upon the concomitant alterations of textures and organs (whether these precede as its causes, accompanying as its coincidences, or as co-effects of some primary cause, or follow as its results) found in the victims of pericarditis. In this particular branch of the subject Dr. Taylor's inquiries stand absolutely alone; and we must express (this is not a mere conventional trick to get rid of the matter, but the announcement of a fact and a verity) our sincere regret that the bulk of our Number prevents us from accompanying the learned author *seriatim* through this division of his subject. Let us content ourselves with an enumeration of the various morbid states observed, and some commentary upon a few of the number. These states, then, were: acute endocarditis, acute carditis, acute pleurisy, chronic pleurisy, pneumonia, acute bronchitis, meningitis, softening of the brain, hemiplegia, acute peritonitis, erysipelas, phlebitis, inflammation and ulceration of the colon, enlargement of the heart, temporary sanguineous engorgement of the heart, old valvular disease, disease of coats of aorta, tubercles in the lungs, emphysema, asthma, œdema of the lungs, serous effusion in pleura, anemia, cirrhosis of the liver, atrophy of the liver, enlargement of the spleen, ascites, anasarca, Bright's disease of the kidneys, chronic ulceration of the stomach, encephaloid cancer of the bladder, &c. Every one of these morbid states receive very full illustration in respect of its diagnosis and symptomatology (more especially in regard to connexion with pericarditis) at the hands of Dr. Taylor.

First, of endocarditis. We have in a previous number (No. XLV, p. 173, January 1847) given in full a passage from Dr. Taylor's paper in the 'Medico-Chirurgical Transactions,' wherein he discusses the question of the possible distinction of the date of an endocardial murmur in a patient seen for the first time, and without cardiac history which can be come at.

We have there shown how Dr. Taylor's experience fails to meet the most difficult part of the subject (and since we wrote the page in question, new difficulties, under a combination of circumstances hitherto unrecorded, have arisen to ourselves, giving yet a further dash of insolubility to the problem), while at the same time we gave him due praise for the excellence of his inquiries, so far as they go. We beg the reader interested in Dr. Taylor, and in men of his stamp of mind, will look back to the Number referred to. But this same endocarditis serves as Dr. Taylor's thesis for more than one interesting discussion. Here, for example, in Case xxx: a woman was cut off with various acute and some chronic ailments; of the former (with which only we have to do) were lobular pneumonia, endocarditis, and pericarditis. The lecturer observes:

"This form of pneumonia, and perhaps this exclusively, is found in cases of purulent infection of the blood, consequent on phlebitis, or occurring in other circumstances. The pus-globules are perhaps arrested mechanically in the capillary vessels of the lungs, and there excite inflammation. Hence the disseminated character of the inflammation in the first instance................These considerations led me to look for some such cause of the pneumonia in this case, and a probable one may be found, I think, in the acute endocarditis seated in the left auricle. Granules of lymph were there found, pus also may have been secreted, and the particles of one or other of these morbid products may have been carried with the current of the blood to the lungs, and there have been arrested."

As far as we are aware, Dr. Taylor is the first propounder of this doctrine, and it is one of no mean importance. He has himself raised a difficulty in its admission in respect of this particular case, namely, that no pus was discovered, though it *may* have been present. But we can get over this difficulty for him. In truth, it is not necessary that *pus* should actually be present as a result of a local inflammation capable of contaminating the blood, and therethrough giving rise to secondary abscesses; the dynamic changes in the fluid, coupled with the circulation of solidified lymph-particles, seem in the existing state of knowledge to be (in all human probability at least) capable, under favouring conditions, of working out the whole series of phenomena ending in peripheral pus-formations. As regards the further evidence, deducible from Dr. Taylor's cases, there are two other examples of fatal endocarditis; in one of these (Case xix) there was lobular pneumonia; in the other (ii) the lungs were not examined. Of seven cases of endocarditis, not fatal, four were attended with pneumonia; in the remaining three (the endocardial inflammation was slight) there were no signs of pneumonia.

In 17 of 38 cases there was acute pleurisy. It is notable that it occurred most frequently in *non*-rheumatic pericarditis; there were 10 examples of pleurisy in 16 cases of this species of pericarditis (62·5 per cent.); 7 in 24 cases of the rheumatic species (29·1 per cent.) The same peculiarity is to be noted in respect of pneumonia: 12 examples of this inflammation occurred in 24 cases of non-rheumatic pericarditis (50 per cent.); 4 examples in 16 cases of the rheumatic species(25 per cent.)

Most valuable remarks appear in the commentary on Case xxiv, on the subject of the causes of hypertrophy with dilatation of the left ventricle. These remarks convey negation of knowledge rather than add to what is presumed to be known; they show that much which is admitted to be certain is problematical, and much which is admitted to be probable is absolutely groundless. But commend to us the man who, like Dr. Taylor, rejoices in such negatives;—can it for a moment be doubted that *no* knowledge is better than *false* knowledge? Thus Dr. Taylor knocks down from its etiological pedestal *diminished elasticity of the coats of the aorta;* certain "physiologists" would make a hard push we know for the restitution of the said diminished elasticity, but if clinical observations say "No," who will be daring enough to say "Yes?" Never has Dr. Taylor known enlargement of the heart "which could be clearly ascribed to *mere nervous excitement* of the organ, however long continued." He most justly affirms that the notion of such enlargement being caused by *unusual muscular efforts* requires to be supported by very different evidence from that which has hitherto been adduced in its favour, before it becomes entitled to the least confidence. Dr. Taylor has no evidence that *habits of intemperance* cause the affection. Again, it is believed that *Bright's disease* acts as a cause of hypertrophy; but the lecturer, wisely calling to mind the numerous instances of advanced disease of the kidney of the kind referred to, in which the substance of the heart is not increased, holds that the influence in question is as yet unestablished. Dr. Taylor thinks that a certain amount of *obstruction in the lungs* may be a cause of enlargement of the left ventricle, as it certainly is of the right. "When the blood," he observes, "for a long time or habitually, cannot pass freely through the lungs—as in emphysema for example—it accumulates in the right cavities of the heart, and causes them to become dilated and thickened. But this obstruction must also be reflected on the capillary circulation; the right cavities of the heart being full, the blood cannot flow into these from the veins, and the veins thus in a state of engorgement cannot receive the blood from the capillaries; hence the necessity for increased efforts on the part of the left ventricle." These ideas are admitted to be speculative, and only to be estimated by an appeal to a large body of facts; it is certain, meanwhile, as Dr. Taylor himself shows, that the left ventricle is sometimes atrophous in cases of emphysema. Hence to revert to our starting-point, the influences really producing hypertrophy of the left ventricle are as yet enveloped by a sufficiently dense cloud of mystery.

In Case xxxvi there was some reason to think that the heart had at one period been in a state of hypertrophy, which had disappeared at the time of death. The case would go then to prove that hypertrophy may be cured by general reduction of the body—for in this instance, the individual had been the subject of cancer of the bladder, and had gradually wasted in mass to a very notable degree. "Could the same result be produced by artificial means? Probably it could (though I have never seen it)." Such are the query and response of Dr. Taylor on the question of the curability by art of hypertrophy of the heart: theory whispers the soft delusion, that the thing is possible; experience cries aloud the hard verity, that the thing is utterly impossible. Which shall we believe? We have shown in a previous Number (January, 1847, p. 180) what Dr. Hope, who represents theory, and Dr. Latham, who represents experience, say on this question: Dr. Hope never failed almost in curing the disease; Dr. Latham never succeeded or knew another to succeed! Verily Dr. Hope was an exquisite specimen of stolid egotism *quoad therapeia;*—and we could name shoulders whereon his mantle hath descended, and sitteth in folds which the wearer judgeth to be full of grace and dignity,—but it is more to the purpose that we record our own experience as being accurately in accordance with that of Drs. Latham and Taylor.

Passing over a mass of materials well worthy of study, we come to the consideration of the treatment of pericarditis; and the first general result of Dr. Taylor's experience on this head is, that the pericardial inflammation attendant on rheumatism is much less fatal, than that which occurs without rheumatism. If, then, one of the class of *ex professo* curers of disease—men privileged above their fellows—raise his modest boast of the success with which *he* treats pericarditis, just ask him (in all probability he will be unable to answer the query, for your true therapeutist soars in a region where such petty matters are unthought of), nevertheless, ask him, whether his cases were of rheumatic or non-rheumatic pericarditis. And then quietly inform him, that pericarditis of all kinds indiscriminately, and pericarditis left utterly to itself—*untreated and uncared for*—does not prove fatal in more than one in six cases.* And how will your therapeutist reply, and how will he act? Why he will utter curses not loud but deep on all statistics and numerical methods that ever were created; he will continue to believe (or to feign to believe) in his providence-sent prowess, and hold forth yet more energetically than ever to gaping youths concerning the all-potency of art in warding off, staying the progress of, or curing completely all manner of evils that flesh is heir to. But no matter for all this; let sober men continue to register experience, albeit that experience tell sad tales of the powerlessness of art; let them be assured that it is only when the amount of that existing powerlessness is generally felt and understood, that improvement in therapeutics may rationally be looked for.

The next general inference deducible from Dr. Taylor's recorded cases, is illustrative and confirmatory of the tenor of the remarks we have just made. In four cases pericarditis got well without treatment, being in one of these instances complicated with dysentery, and in another with endocarditis. In a fifth case the pericarditis, which got well under treatment, would probably have got well without any, seeing that the patient was not submitted to any until the twelfth day of the disease. Dr. Taylor comments, as follows, on the case (xxx) of pericarditis associated with dysentery above referred to:

"It deserves to be remarked that the pericarditis certainly, and the inflammation and ulceration of the colon very probably, had escaped detection, and consequently had not been treated. Both diseases had been very severe; yet both, I think, had been cured by the powers of Nature. The patient died, but, as it appears to me, of another disease; and I think there is every reason to believe that, notwithstanding the absence of treatment for the inflammations in question, life would have been spared, although perfect health could not have been restored. Such cases are very common. I have repeatedly seen pneumonia (one of the most fatal of all internal inflammations) and pleurisy terminate favorably without any active treatment, their existence having been overlooked until their course was nearly run. Surely these and similar facts should teach us a little more caution in assigning so large a part to ourselves, and so small a part to Nature, of the credit that is attached to the favorable termination of cases of even severe inflammatory diseases."

The more important plans of treatment illustrated by Dr. Taylor's cases

* Louis, Mém. Anat. Pathol., p. 289. Paris, 1826.

are those by general and local bleeding, and by mercurials. We do not find anything very plainly novel brought out in connexion with the two former; but the inferences drawn by the learned author concerning the power and influence of mercury, are most singularly valuable. On the general subject of mercurial treatment in membranous inflammation of the heart, we have spoken in the Number of this Journal already more than once referred to. We there submitted the statements and facts published by Dr. Latham on this question to examination;—and it proved an easy task, not only to show that that physician's *general* high estimate of the powers of the mineral was not borne out by his *particular* facts, but that he himself, in the course of his examination of those facts, was in common honesty compelled to soften somewhat the tone of almost worship with which the influences ("antiphlogistic and reparatory") of mercury were in their general bearings set forth. In the present place, then, we shall simply lay before the reader a series of inferences, supplied by Dr. Taylor's cases, illustrating the utility or non-utility of mercury in pericarditis, and some other inflammations.

1. Ptyalism was not followed by any abatement of the pericarditis in *twelve* cases (i, ii, iii, v, vi, xi, xiii, xiv, xv, xvii, xxii, xxiii).

2. In *one* case ptyalism was followed by speedy relief (vii).

3. In *two* cases (ix, xxvii) ptyalism was followed by a diminution, and then gradual cessation of pericardial murmur.

4. In *one* case pericardial murmur had been diminishing for some days before, and it ceased soon after ptyalism was produced (xvi).

5. In *one* case pericarditis and pneumonia both increased in extent and intensity after ptyalism (xxi).

6. In *four* cases pneumonia supervened after the establishment of, and therefore was not prevented by, ptyalism (iv, v, xii, xv). [Was it CAUSED by it?]

7. In *three* cases endocarditis supervened after ptyalism (v, x, xiv).

8. In *six* cases ptyalism was followed by pericarditis (xi, here the mouth was very sore at the time, and the pericarditis continued sixteen days; xii, xxi, xxiii, here the two things were nearly of simultaneous origin; xxiv, xxvii).

9. In *one* case ptyalism could not be produced, and yet the pericarditis went on favorably (xx, mercury was given for nineteen days).

10. In *two* cases ptyalism was followed by extensive pleuritis (xxiii, xxiv).

11. In *one* case ptyalism was followed by erysipelas and inflammation of the larynx (xxvii).

12. In *two* cases rheumatism continued long after ptyalism was produced (xvi, xvii).

The importance of these propositions need not be insisted on,—they very clearly show that current opinions on the powers of mercury require revision. The question of the issue of pericarditis treated with mercury, and of pericarditis not treated with mercury, cannot be solved by these cases for several obvious reasons; but these cases may be used as elements for its decision by future inquirers—Dr. Taylor's subscription in the cause. The same may be said of their applicability in reference to the question of the comparative influence of mercury in rheumatic and in non-rheumatic cases of the disease.

So far we have been unassisted, in our examination of Dr. Taylor's cases, by any published analysis of them by the author himself. It had been his intention to undertake this labour (and unquestionably upon a more minute and extended plan than that followed in the preceding pages by ourselves), but circumstances appear to have occurred preventing its fufilment. The author has, however, published an analysis of his experience in respect of a special question, the etiology of pericarditis,* and of this we proceed to lay a brief notice before our readers,—premising that modern medical literature scarcely to our knowledge (and we have full cognisance of the labours of Louis and his followers) contains a single Essay which may reasonably be said to vie with this in closeness of argumentation and precision of induction.

The author does not profess in this paper to investigate *all* the causes of pericarditis. He has first inquired what were the causes actually observed in all the instances of the disease which had fallen under his notice; and, in the next place, investigated more in detail the frequency, both absolutely and relatively to each other, of each of these observed causes, as well as some other of the circumstances connected with them. And, finally, he has examined, incidentally, the influence of the same causes in producing inflammation of other internal organs, both in connexion with and independently of pericarditis.

The cases of acute and severe pericarditis, examined in the paper and added in a note, are 38 in number. Of these

20 occurred in the progress of acute rheumatism;

1 was probably rheumatic;

10 were complicated with Bright's disease;

1 was complicated with some renal disease and empyema;

1 was complicated with some renal disease, but probably not Bright's disease;

1 was complicated with double chronic nephritis and encephaloid cancer of the bladder;

2 *may have been* complicated with Bright's disease;

1 was complicated with malformation of the heart and cyanosis;

1 proceeded from extension of inflammation from contiguous textures.

These cases of severe pericarditis may be conveniently divided into two groups: first, of those occurring in persons previously in good health or actually labouring under some acute disease; and secondly, of those occurring in persons in bad health or labouring under some chronic disease. A remarkable and important difference is discoverable in these two groups in relation to the causes of the disease. For it appears that of 16 cases belonging to the first group, all were complicated with acute rheumatism, and none (so far as is known) with Bright's disease; while of 13 cases, belonging to the second group, only one was complicated with acute rheumatism, whereas fully two thirds *were known* to be associated with Bright's disease, and all of them may have been so associated. The chief causes of pericarditis are therefore inferribly two in number, namely, acute rheumatism and Bright's disease.

Dr. Taylor next enters into some considerations intended to show that these two diseases own their power of inducing pericarditis to the same kind of ultimate cause, namely, an alteration in the composition of the

* Med.-Chir. Transactions, vol. xxviii.

blood; but he does not attempt to determine whether that alteration be essentially the same in the two diseases. And further, if (he argues) it be assumed that in the single case of pericarditis, associated with cyanosis, the acute disease depended upon the state of blood existing in the chronic affection, it will follow that only two causes of the membranous inflammation of the heart were observed in the 38 cases under consideration,—namely, a morbid condition of the blood, and extension of the inflammatory process from a neighbouring texture.

The author next examines his cases of adhesion of the pericardium and of "white spots" on its surface, with reference to the causes of the inflammation producing them. This examination leads to the inference that, in every case in which any information was obtained on the point, there had previously been either acute rheumatism or pleurisy, or there was found, actually existent, either Bright's disease or some other disease of the kidney.

Dr. Taylor proceeds in continuation to examine with more detail the two affections mentioned, in regard of their agency in producing pericarditis,—and (first) *acute rheumatism.*

I. The frequency of acute rheumatism as an observed cause of pericarditis was, as already stated, 75 per cent. The complementary inquiry into the frequency of the cardiac inflammation in the course of acute rheumatism remains to be made. Now it appears that among 75 cases of acute rheumatism, treated by the author in University College Hospital, there were 37 (or about half) in which there was disease of the heart of some kind or degree; in the others there was probably none. Among these 75 cases of rheumatism there occurred 6 instances of acute pericarditis of considerable severity, besides 2 of very slight character. The proportion of the former therefore was 1 in $12\frac{1}{2}$ cases. In the same 75 cases of rheumatism there were 32 cases of valvular disease of the heart, either old or recent, besides 2 known to be recent. There was therefore 1 case of valvular disease in about every 2 cases of rheumatism.

Comparing these results with the statements of various writers upon the same subject,* Dr. Taylor draws the two following conclusions: 1st. That acute inflammation of the heart has occurred less frequently as a complication of acute rheumatism in his experience, than it has been *believed* to occur in the experience of those writers whose opinions seem to have been most generally adopted by the profession. 2d. That the frequency of cardiac inflammation, even in his cases, has been such as abundantly to show the great influence of acute rheumatism in its production. An attempt is then made by our laborious author to ascertain the real amount of the difference (with its causes) between his own observations and those of the writers referred to. The result, as respects most of those writers, may be briefly stated as follows:

* Some of these statements being totally unsupported by numerical estimates, are obviously not worth the paper they cover, and might have been omitted by Dr. Taylor. But we thank him for placing once again before the world (many a time and oft have we pondered in bewildered wonderment over the original phrases) the notorious dicta of Dr. Hope on this subject. Dr. Hope, having recorded his opinion, that in *about one half* of all cases of rheumatism either neglected or "inefficiently treated" (which, being interpreted, means treated on any other plan than the particular one he fancied and patronized), complacently adds: "I think about *one case in twelve* would be the maximum in MY practice." ! This absurdity we thank Dr. Taylor for recording in an additional place, because it may serve to prevent some youths, possessed of the loose reasoning faculty of Dr. Hope, from entering upon a therapeutical career such as his. Even Dr. Hope did not *begin* with such feats as the above: let them look in time *to what they might come.*

First (in connexion with *pericarditis*). In those instances, in which such data have been given as enable us to compare *similar* cases, the results are very nearly the same. In various instances, however, no comparison can be fairly made, either from the want of figures, from the fact that cases of endocarditis and of pericarditis are mixed up together, or from a great difference in the age of the patients. Secondly (in connexion with *endocarditis*). The discrepancy is much greater than in cases of pericarditis; and one of the chief causes of the difference appears to be, that most writers have given the proportion of cases of valvular disease, in acute rheumatism, in such a manner as to imply (when this is not actually and directly stated) that they were all cases of acute disease,—omitting, in other words, to distinguish the proportion of these valvular affections which were of older date. (Vide on this point our Number for January, 1847, pp. 172 et seq.)

The proportion of cases of valvular disease of all dates observed by the author is nearly the same as that observed by the chief writers referred to; but he attempts to show (and succeeds completely), in the first place, that the greater number of these are examples of *old* valvular disease; in the second place, that at all events in most cases it is very difficult to distinguish when the disease is recent and when old; and, in the third place, that acute endocarditis is less frequent than acute pericarditis in rheumatism.

The frequency of disease of the heart in chronic rheumatism is now inquired into and compared with that in acute rheumatism; and from this inquiry it appears: (*a*) that the total number of cases of morbus cordis (old and recent) is nearly the same in the two kinds of rheumatism; (*b*) that acute inflammation of the pericardium and of the endocardium is much more common in cases of acute than of chronic rheumatism.

Dr. Taylor next turns his attention to the circumstances favoring the supervention of cardiac inflammation during the course of acute rheumatism. (*a*) The doctrine of *metastasis* has probably no supporters now in any quarter of the medical globe; and it is satisfactory to find that the cases analysed by their author give warranty to the prevalent views. Metastasis did not occur in any one of the cases observed. Dr. Taylor hence infers that metastasis is not of the ordinary—nor even a frequent—mode in which rheumatism produces the heart-affection. It does not, however, follow from these facts (he urges) that metastasis never takes place; and an attempt is made to show that its occasional occurrence is both consistent with theory and established by observation,—an attempt in which the author appears to us less felicitous than usual. In connexion with this question Dr. Taylor refers (1) to some cases of rheumatism in which the inflammation of the heart appeared before that of the joints; (2) to certain cases of what has been termed "rheumatic fever without arthritis;" (3) to one of his own cases, in which he thinks it probable [and we think so too] that there may have been acute rheumatism, and in which there was pericarditis, but no affection of the joints throughout. (*b*) As respects the particular influence of *particular forms* of rheumatism; all Dr. Taylor's cases of rheumatic pericarditis occurred in connexion with the *fibrous* as distinguished from the *capsular* (Macleod) form of the disease. It must not be forgotten, however, that the former variety is much more common than the latter. (*c*) *Intensity of the rheumatism.* From the cases examined it appears to result that the *violence and fatality* of rheumatic pericarditis are generally greater in the cases in which the accompanying rheumatism is very acute than in those in which it is subacute. Whether pericarditis be more *frequent* in the more severe than in the less severe form of rheumatism, the author's cases, he confesses, do not enable him with confidence to determine; as far as they go, however, they are opposed to the notion of the frequency of the one being in the direct ratio of the severity of the other,—for three fourths of the examples of rheumatic pericarditis occurred in subacute rheumatism. (*d*) *Stage of the rheumatism.* In more than one half the cases of rheumatic pericarditis the affection of the heart appeared on or before the fourth day of the disease. With one exception, the pericarditis did not appear sooner in those cases in which it was very severe than in those in which it was much less severe. (*e*) *Influence of repeated attacks of rheumatism.* In the cases examined pericarditis was found to be both more frequent and more severe in the first than in subsequent attacks of rheumatism. (*f*) *Previous disease of the heart.* Ten out of fifteen patients had no previous disease of the heart; and among these were found all the most severe cases of pericarditis. (*g*) *Age.* Of 15 patients 9 were aged 20 or under, 5 from 20 to 26, and 1 above 40. (*h*) *Sex.* Of 15 patients 9 were males and 6 females. Rheumatism is, however, more common among men than women. (*i*) *Influence of venesection.* 12 of these 15 patients had not been bled before the pericarditis appeared; the remaining 3 were bled, 1 eleven days, 1 five days, and 1 three days before the pericarditis supervened.

Even Dr. Taylor must have his hypotheses. Here is an outline of his theory of the manner in which rheumatism produces pericarditis. The cause of acute rheumatism is probably the presence of some morbid matter in the blood, which has an especial affinity for the fibrous and fibro-serous tissues of the body, and which, by fixing itself in one or more of these, induces various local inflammations. The similarity of the structures implicated is probably the reason why rheumatic pericarditis or endocarditis often occurs at the same time with, or succeeds to, rheumatic inflammation in the joints (Bouillaud), just as rheumatic inflammation in one joint occurs with or succeeds to that in another. And the heart is more frequently [??] and more severely affected in severe cases of acute rheumatism for the same reason that more joints are affected, and these more severely affected, and more fever present, in such cases. And the efficient cause of such greater severity may not improbably be a greater abundance of the materies morbi in the blood. Now we cannot, we confess, concede to this theory the quality of close inference; and in some of its elements it is deliberately defective. Observe Dr. Taylor arguing that the heart is more frequently affected in severe than in mild cases; though his facts, as far as they go (see last page), positively demonstrate the reverse. But if he fail, he may console himself with the reflection that this is a matter in which *le plus fort perdrait son Latin.*

II. Next the author enters upon a detailed examination of Bright's disease as a cause of cardiac membranous inflammations.* It has already

* Since the production of Dr. Taylor's paper, exhibiting, as it does, clearly and for the first time, the *powerful influence* of Bright's disease in generating pericarditis, many individuals have undergone abrupt opening of the eyes as to the extensive and accurate information they themselves already possessed on the point. As though the fact, vaguely known even by the most ignorant, that among the been seen that in 13 (or more than one third) out of 35 cases of pericarditis Bright's disease was the only assignable cause of the inflammation. In respect of the frequency of pericarditis and endocarditis in Bright's disease hear Dr. Taylor's facts.

1. In the bodies of 50 patients who had either died of Bright's disease, or who were ascertained to have this disease in an advanced stage—

Acute pericarditis was found in 5, or in 1 out of 10.

Acute endocarditis was found in 4, or in 1 out of 12.

2. On the other hand, in 142 bodies in which the kidneys were not affected with any appreciable disease—

Acute pericarditis was found in 4, or in 1 out of 35.

Acute endocarditis was found in 2, or in 1 out of 71.

Pericarditis and endocarditis therefore, being four times more frequent in fatal cases of Bright's disease than in fatal cases without renal disease, it seems clearly to follow that the influence of Bright's disease in producing these inflammations is unquestionable and great.

3. From a comparison of the frequency of other internal inflammations in fatal cases of renal disease, and in fatal cases without renal disease, it appears : (*a*) that the proportional number of acute internal inflammations (exclusive of those of the heart) is twice as great in the series of cases with renal disease as in that without such disease,—the numbers being respectively 96 and 42 per cent. (*b*) That the proportion of patients likewise, among whom these inflammations were distributed, is greater in the former than in the latter series of cases, the numbers being respectively 60 and 36 per cent. Hence the safe inference is drawn that Bright's disease has a great tendency to produce other internal inflammations besides those of the heart.

4. A further examination of the same facts shows that the relative frequency of various internal inflammations is different in fatal cases of Bright's disease and of other diseases taken indiscriminately. The following inflammations (those inquired into by the author in connexion with the present point) are arranged in the order of their frequency, as they were calculated to be due to renal disease, or to the causes operating in other fatal diseases. (*a*) *Inflammations due to renal disease.* Cerebritis, pneumonia, pleuritis, pericarditis, endocarditis, meningitis, peritonitis. (*b*) *Inflammations independent of renal disease.* Pleuritis, pneumonia, peritonitis, meningitis, cerebritis, pericarditis, endocarditis.

5. The numbers given in the author's paper allows us to calculate the comparative tendency to produce internal inflammations exhibited, on the one hand, by the sum of causes operating in fatal cases of Bright's disease,—and, on the other hand, by the sum of causes present in fatal cases without renal disease. The result is displayed in the following tabular arrangement.

secondary serous inflammations of the renal disease, sparse examples of pericarditis might be found, could (except by the most addled of brains) be considered anticipatory of the discovery that Bright's disease (in its advanced, as distinguished from its early, stages) is as active a cause of pericarditis as acute rheumatism ! Hear the remarkable profession of faith put forth by Dr. Latham on this point : "My own experience of pericarditis is mainly derived from what it is, as an accompaniment of acute rheumatism. I have seen the disease, indeed, under other circumstances ; but it has been very seldom ; so seldom, indeed, that I have little acquaintance with other conditions, external or internal, conducing to it. *I can neither tell whence to look for it, nor when to expect it,* except when it occurs as a part of acute rheumatism.........Separate from acute rheumatism, even the practice of a large hospital does not present me *with more than an instance or two of it in several years.*" (Lectures on the Heart, vol. i, p. 136.)

*Bright's disease produces*

*a.* Endocarditis *almost* 5 *times as often as* all other causes put together.
*b.* Cerebritis *fully* 3½ *times as often as* ,,
*c.* Pericarditis ,, 2½ *times as often as* ,,
*d.* Pneumonia ,, *just as often as* ,,
*e.* Pleuritis ,, ¾ *times as often as* ,,
*f.* Meningitis ,, 3 *times less frequently than* ,,
*g.* Peritonitis ,, 100 *times less frequently than* ,,

In instituting his next inquiry, namely, into the comparative power of acute rheumatism, and of Bright's disease in producing pericarditis and other internal inflammations, the author meets at the threshold with a difficulty, in the fact that one of these diseases is acute and seldom fatal, the other chronic and generally fatal. Dr. Taylor considers that the best way of steering clear of this difficulty is by comparing *fatal* cases of Bright's disease with ordinary cases of acute rheumatism. If the object were to ascertain the proportion of cases, in which traces of *previously existing* inflammation were found, this method would be decidedly objectionable; because the one disease, having run a much longer course than the other, would have had much more time than the latter to produce any inflammation, which it had the power to produce. But if cases of *actually existing* inflammation alone be counted, the same objection (or certainly not by any means to the same extent) does not exist; and the result should not (we agree with the author) be far from the truth. It appears, then, that of

75 cases of *acute rheumatism,* 8 were complicated with pericarditis (1 in 9½).

50 cases of *fatal Bright's disease,* 5 were complicated with pericarditis (1 in 10).

Hence Bright's disease, *in the advanced stage,* and acute rheumatism appear to have caused acute pericarditis in a very closely equal proportion of cases. An examination of 20 cases of *old* adhesion of the pericardium, however, shows (what the considerations already put forward might have led us to anticipate) that *old* adhesions of the membrane were produced twice as often by Bright's disease as by previous attacks of acute rheumatism.

From considerations remarkable for their sustained closeness and sagacity, but for which we must refer our readers to the original, Dr. Taylor infers that acute rheumatism has a greater tendency to produce pericarditis than has Bright's disease in its earlier stages; and consequently that the tendency of Bright's disease to induce pericarditis, and probably also other internal inflammations, increases in proportion as the affection of the kidney is more advanced. This inference (which had on imperfect and theoretical grounds been admitted by others) it is pleasurable to find established, as it is established, in these pages.

In conclusion, Dr. Taylor makes some remarks upon the probable occurrence of pericarditis in other blood-diseases besides those observed by himself; and likewise on the importance of the constitutional or predisposing causes of inflammation, as distinguished from the exciting causes. To these we invite the reader's attention.

The esteem in which we hold the contributions of Dr. Taylor to the history of pericarditis, appears not only from the closeness with which we have analysed them, but from the simple fact of our having noticed them at all in the pages of this Journal. As our readers are aware, it is not our habit to cull papers from periodical literature, and make for ourselves from the sum of these the basis of an analytical article. The absolute value of the original productions, and the hope that Dr. Taylor's example would lead others to engage in pure clinical observation (apart from chemistry and micrology gone mad) led us to swerve from our usual habits. We trust Dr. Taylor will give us more of his "Contributions;" but whether he do or not, he has established for himself the highest character the physician can aspire to—that of a sagacious and an *honest observer*.

